# Equivocal diagnostics: Making a ‘good’ point-of-care test for elimination in global health

**DOI:** 10.1177/03063127241246727

**Published:** 2024-04-23

**Authors:** Alice Street, Emma Michelle Taylor

**Affiliations:** University of Edinburgh, Edinburgh, UK

**Keywords:** diagnostics, neglected tropical diseases, innovation, product development, global health

## Abstract

What is a diagnostic test for? We might assume the answer to this question is straightforward. A good test would help identify what disease someone suffers from, assist health providers to determine the correct course of treatment and/or enable public health authorities to know and intervene in health at the level of the population. In this article, we show that what a specific diagnostic test is for, the value it holds for different actors, and what makes it good, or not, is often far from settled. We tell the story of the development and design of a rapid antibody test for onchocerciasis, or river blindness, tracking multiple iterations of the device through three configurational moments in the framing of onchocerciasis disease and reshaping of the global health innovation ecosystem. Efforts to build that ecosystem for diagnostics are often premised on the notion that public health needs for diagnostics are pre-given and stable; the challenge is seen to be how to incentivize investment and find a customer base for diagnostics in under-resourced settings. By contrast, we show that for any disease, diagnostic needs are both multiple and constantly in flux, and are unlikely to be met by a single, stand-alone product. In the case of the onchocerciasis Ov-16 rapid test, the failure to recognize and address the multiplicity and instability of diagnostic needs in the innovation process resulted in the development of a rapid point of care test that might be manufactured, procured and used, but is unloved by public health experts and commercial manufacturers alike. The equivocal value of the onchocerciasis rapid test, we suggest, reveals the inadequacy of the current global health innovation ecosystem for developing diagnostic ‘goods’.

New framings of diseases can dramatically transform their fortunes in global health, propelling them onto priority lists and spurring investments in the development of drugs, vaccines and diagnostic tools (Aronowitz, 2008; Leach et al., 2010; Wilkinson, 2017). The US military’s reframing of Lassa fever in West Africa as a biosecurity threat in the early 2000s prompted investment in the development of new diagnostic tools where the previous efforts of activists operating in a more ‘humanitarian’ frame had failed (Lakoff, 2010; Wilkinson, 2017). Likewise, the formal designations of Ebola, Zika and Covid-19 as a Public Health Emergency of International Concern (PHEIC) galvanized efforts to accelerate the development of new diagnostic tests (Kameda et al., 2021; Kelly et al., 2022). The resurgence of global health disease eradication and elimination campaigns since the early 2000s have been similarly instrumental in the reframing of Neglected Tropical Diseases (NTDs) as global health priorities (Smith & Taylor, 2013) and as candidates for diagnostic development (Taylor, 2019).

This article tells the story of a diagnostic test developed for the purpose of assisting elimination efforts for onchocerciasis, also known as river blindness, the second leading infectious cause of blindness world-wide and targeted by the WHO for elimination through ivermectin mass drug administration (MDA) (WHO, 2010). On the one hand, this is a story of successful global health innovation: a rapid test for an NTD that was developed and commercialized against the odds. On the other hand, as we show, both the test’s manufacturers and its users remain equivocal about the value of the test in its current form, and its commercial and public health futures are less than certain.

At the heart of this story is a protein, the Ov-16 antigen, discovered as a biomarker for onchocerciasis in a research lab at the US National Institute of Health in the late 1980s. Since then, the antigen has been immobilized on the reagent strips of multiple rapid diagnostic tests, variously developed and manufactured in Australia, USA and South Korea, with the purpose of enabling the diagnosis of onchocerciasis in settings without access to a laboratory, a ‘conjugation’ (Lezaun, 2018) of disease and biological technology enabled in part by the expectation that candidates for elimination goals should be ‘tool-ready’ (Dowdle & Cochi, 2011). The recent history of onchocerciasis disease cannot therefore be easily extricated from the history of the Ov-16 antigen as a diagnostic infrastructure, and vice versa.^1^

Developing effective diagnostic tests for neglected diseases is often portrayed as a problem of market failure, the essential problem being the lack of industry investment in the development of products for unprofitable markets. Indeed, as we show, the commercial development of an Ov-16 onchocerciasis test was only made possible by intensive efforts to build a unique kind of global health ‘market’; one in which both ‘investors’ and ‘payers’ consist of philanthropic donors, international organizations and bi-lateral development agencies, rather than venture capital firms and end users. Yet, by tracing the meandering biography of the Ov-16 rapid test, we find that the challenges involved in achieving success as a global health technology go well beyond the question of commercial profitability. As we show, whether or not an onchocerciasis Ov-16 test is good, and what precisely it is good *for*, have been the subject of ongoing negotiation, contestation and controversy, often long after a particular test has entered the market. Diagnostic needs for onchocerciasis have proved as difficult to pin down as diagnostic means have been to develop, revealing that public health needs do not precede technological solutions and efforts to redress market failure so much as they are entangled with them. The economic value of a diagnostic test and its public health value are equally unstable. Our focus on efforts to define and align those different forms of value in the process of product development, provides insight into the peculiar status of the Ov-16 rapid diagnostic test (RDT) as a technology that has neither failed nor flourished, that is commercially available and yet, we argue, is not enthusiastically loved by either its manufacturers nor its consumers.

Social studies of technology have often told stories of success or failure, examining the social, historical and political contingencies that lead some technologies to be scalable and to travel, and others to fail to fulfil expectations or to accrue support at the crucial moments (Akrich, 1992; Latour, 1996; Law, 2002). But equivocal technologies like the onchocerciasis RDT are arguably more common than are either of these absolutes. Such technologies may be difficult for either social scientists or public health professionals to ‘love’ (De Laet & Mol, 2000; Redfield, 2016), but their equivocal status might nonetheless provide a unique perspective on processes of value generation in global health R&D, and in particular the challenge of aligning public health and economic value regimes in a single technological device (Engel, 2020; Street, 2023).

Taking a broadly biographical approach, our analysis focuses on three ‘configurational movements’ (Hyyasalo et al., 2019) in which new framings of onchocerciasis took centre stage, new actors came into the orbit of the rapid test, and new technological capacities became possible. In each of these movements, we show, public health needs were imagined to be met by testing products based on Ov-16 in specific ways. The first of these configurational movements relates to the development in the late 1990s by a small Australian biotech start-up of a new rapid onchocerciasis test based on the recombinant Ov-16 antigen, and the shelving of that product after several company acquisitions, as market demand failed to materialize. This failure, we show, was a direct result of the framing of onchocerciasis as a disease that requires ongoing ‘control’; a goal that was adequately supported by existing diagnostic methods and did not need a rapid antibody test to be achieved. Second, we follow the development of a new iteration of an Ov-16 test through a public–private partnership in the early 2010s, this time backed by funding from the Bill & Melinda Gates Foundation (BMGF) and the promise of there being a donor-led market bolstered by elimination goals set by the WHO. Last, we describe the incremental acquisitions through which the Ov-16 test ended up in the hands of a large US pharmaceutical firm, Abbott, which now holds what could be construed as an effective monopoly over the commercially available diagnostics for onchocerciasis and several other NTDs. The future of onchocerciasis’s conjugation with the Ov-16 rapid test hangs in the balance; while the test continues to be available to order, and is to some extent appreciated as a rare commercialized tool in a sparse market, it appears neither to be wholeheartedly loved by its manufacturers nor its users. Ultimately, we argue, the creation of this equivocal test was the result of a model of diagnostic innovation that not only struggles to secure the stable supply of global public goods, but is also unable to generate consensus around what makes those goods ‘good’. We conclude with a discussion of the issues that this raises for efforts to build more equitable relations into global health innovation processes.

## The diagnostic good

In tracking the construction and alignment of the onchocerciasis RDT as a commercially viable commodity and a public health good, we build on two critical approaches to global health product development. The first, which we term a ‘distribution’ approach, focuses on the question of who benefits from the development of global health commodities. Scholars working in this vein have examined the flows of funding and profit involved in efforts to stimulate industry interest in areas of global health that are deemed unprofitable, such as NTDs. Scholars have pointed out that arrangements that are intended to overcome industry hesitation, like product development partnerships or advanced market commitments, do so by shifting the risk burden of product development from the private to the public (or philanthropic) sphere, while resulting profits flow almost entirely to the firms that manufacture them (Buse & Harmer, 2004; Graham, 2019; Lazonick & Mazzucato, 2013; Light & Lexchin, 2012; McGoey, 2014; Richter, 2004). For example, pharmaceutical and biotech firms contributed little ‘added value’ to Ebola Vaccines, whose molecular foundations were originally discovered and developed with public funds and academic effort. And yet, through their ownership of the means of mass production, those firms were able to claim ‘pseudo-authorship’ of resulting products and the ensuing profits (Graham, 2019).

Another take on ‘distribution’ has been to focus on global inequities in access to life-saving medical technologies generated by current intellectual property regimes. Accounts of medical product development during the COVID-19 pandemic, for example, focused almost universally on the ways in which international legal frameworks of intellectual property ownership, which are designed to protect commercial interest, locked poor countries out of markets for life-saving diagnostic tests, drugs and vaccines (Banerjee, 2021; Harman et al., 2021; Kirksey, 2021; Sparke & Williams, 2022; Su et al., 2021).

However, a focus on the distribution of profit or commodities leaves little space for discussion and analysis of whether those goods were actually any good. As Redfield (2018) has pointed out for vaccines and drugs, it is hard to argue against life-saving technologies. It is perhaps for this reason that critiques of global health R&D regimes have tended to focus on the morally ambiguous conflation of public goods with private interests, rather than on the question of whether and why particular products are deemed to be needed. And yet, while the ‘need’ for diagnostics, drugs and vaccines can seem incontrovertible in principle, the story of the onchocerciasis RDT that we tell below shows that the translation of an abstract need for elimination into a specific need for diagnosis, and the translation of a general need for diagnosis into the need for a specific diagnostic product are neither inevitable nor smooth.

In a second analytic approach to global health product development, which we term a ‘health-systems approach’, scholars have focused attention on medical devices themselves and examined the problems inherent in technology-driven global health frameworks. These frameworks often operate through vertical, disease-focused programmes, and fail either to acknowledge long colonial and post-colonial histories of neglect or to contribute to the improvement of routine healthcare and infrastructure (Biehl & Petryna, 2013; Packard, 2016). Scholars working in this approach have argued that the narrowing of the global health imagination to the development of quick technical fixes does little to assist and strengthen chronically neglected health systems in the Global South (Adams, 2016; Graham, 2016; Parker & Allen, 2014; Storeng, 2014), and that the minimalist, mobile technologies often favoured by such programmes, such as bed nets (Beisel, 2015), rapid diagnostic tests (Beisel et al., 2016), or water filters (Moran-Thomas, 2015), frequently fail to live up to expectations that they patch chronic infrastructure gaps (Redfield, 2018). Such studies have exposed the short-sighted nature and often damaging effects of technological optimism. However, there is also a risk that such an approach can lead to the uncritical denigration of technology in general (Beisel et al., 2016) while sliding into nostalgia for a twentieth century model of the welfare state that was never fully realized, and that conjures the ‘health system’ as an ideal, without giving it definition or specificity (Geissler, 2015; Redfield, 2018).

Building on both distribution and health-systems approaches to global health technologies, we likewise adopt a sceptical stance toward partnership-models of product development and technology-driven global health agendas. Yet we also seek to extend these critiques by examining the ‘practices of valuation’ (Antal et al., 2015) by which diagnostic products are constructed as public health *goods*, and probing how distinct value regimes, for example those driven by concerns about profit, security, or humanitarianism, might become aligned (or not) in the process of product development and deployment (Cross & Street, 2022; Engel & Wolffs, 2020; Street, 2023). As Engel and Krumeich (2020) have pointed out, valuations of what makes a diagnostic test ‘good’—for example their simplicity, rapidity and accuracy—vary between diverse multiple actors and across a product’s life cycle. For a test to be successful requires significant effort by developers and implementers to align distinct values, settings, and temporalities and ‘atune’ a test to its contexts of use, often in the context of social and scientific uncertainty (Engel, 2020; Engel & Wolffs, 2020; Kameda et al., 2021; Street, 2023).

Our ‘biography’ (Hyysalo et al., 2018; Kopytoff, 1986) of the Ov-16 RDT traces its rocky and circuitous trajectory through multiple partnerships, mergers and acquisitions, focusing on how that path shaped both the test’s technological capacities and the value it held for heterogeneous actors in an emerging moral economy of global health R&D (Kelly et al., 2022; Lezaun, 2018; Lezaun & Montgomery, 2015; Mei, 2017). We construct these configurational movements from a combination of semi-structured interviews with people working in onchocerciasis policy, in programme delivery and research, and in the broader global health diagnostics community; fieldwork at conferences related to NTD policy and research; and a review of relevant policy and organizational documents and reports and a scoping review of the literature on onchocerciasis diagnosis (Taylor, 2020).

## Ov-16 shelved

In the late 1990s, a small biotech start-up focused on humanitarian solutions, ICT Diagnostics, was launched in Sydney, Australia. ICT’s founder, Anthony Smithyman, had grown up in Malawi and was interested in the potential for new monoclonal technologies, which were taking the life sciences world by storm, to address the challenges of tropical diseases in Africa. Smithyman had acquired the rights to a new cardboard immunochromatographic (ICT) rapid testing platform, which used a simple test-strip format that harnessed the immunological response of antibodies to target proteins to detect the presence of pathogens in bodily fluids. As Smithyman told us:

This little cardboard test was so convenient for field testing. You could suddenly see that you could go out and do these tests. You didn’t need a mobile laboratory, you didn’t need anything, you just needed the test and a bit of blood. So that was the starting point and we went from there.

The recent commercialization of rapid diagnostic tests for malaria, based on similar technology to ICT’s platform, had suggested to Smithyman that untapped commercial opportunities existed in the diagnostics sector for tropical diseases, and he believed that a latent market for portable, easy-to-use diagnostic devices could be found in the large-scale disease control campaigns led by international organizations like the WHO and the Rockefeller Foundation. At the time, there was no seed funding from BMGF (which did not yet exist), or social-investment groups, and it was unclear where investment for such a venture might come from. Smithyman penned an article that was published in the Sydney local press with the title ‘Rapid diagnostic tests for $1’, attracting the unlikely interest of a group of Australian real-estate men who had made their money building large factories on the fringes of Sydney and who agreed to invest in the outfit.

Commercial success came early, with the development of an RDT for malaria, and a test for lymphatic filariasis (LF) in the late 1990s that was quickly picked up by the newly established Global Programme to Eliminate Lymphatic Filariasis, and rapidly became a mainstay of LF mapping activities in support of disease elimination goals (Mearns, 1996; Weil et al., 1997). Buoyed by this early success, the team immediately cast around for another infectious disease candidate for their testing platform. A virologist on the team, Gary Weil, happened to know Thomas Nutman, a parasitic disease specialist at the National Institute of Allergy and Infectious Diseases, National Institute of Health (NIH), whose team had first identified Ov-16 as an *O. volvulvus* specific protein (Lobos et al., 1991). Nutman agreed to supply the laboratory manufactured Ov-16 antigen for the purposes of enabling the development of a rapid test for onchocerciasis.

At this point, there was no obviously articulated demand for a rapid onchocerciasis test from the global health community, and the specific use case for the test was undetermined. Instead, the decision by the ICT team to launch into the development of an onchocerciasis test was largely opportunistic, driven by pre-existing academic research networks and scientific curiosity rather than by a specific demand from global health organizations and experts. Nonetheless, the team moved quickly. In a matter of weeks, they had a working prototype of the card test to detect IgG4 antibodies to recombinant antigen Ov-16.

**Figure 1.**
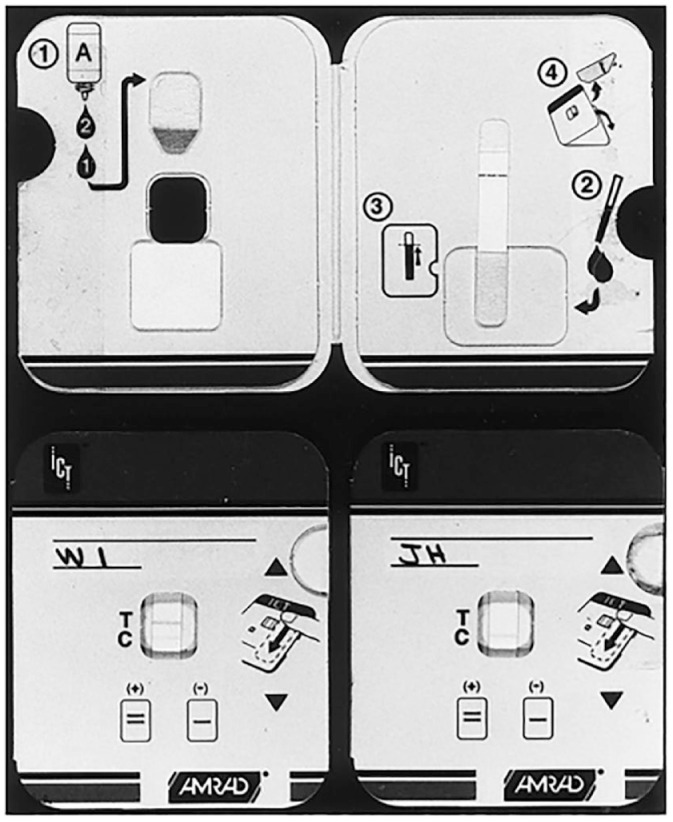
AMRAD ICT card test for onchocerciasis (Weil et al., 2000, p. 1797).

Nutman and Weil went on to trial the prototype in their labs, using stored source material, with Nutman also evaluating the test in travellers returning from onchocerciasis-endemic areas, in a clinic held at NIH. The results of these studies were published in 2000, and reported ‘very good sensitivity’ and ‘excellent’ specificity (Weil et al., 2000). These results were later confirmed by the performance of the Ov-16 card test in field validation studies in Burkina Faso and Côte D’Ivoire (Lipner et al., 2006). The research team acknowledged that, as an antibody test, the RDT was unable to differentiate between past and active infection. Nevertheless, they proposed that the card test might still be used to detect ongoing transmission (and therefore also track the halting or stalling of transmission) if used to test children. And, according to the authors, the easy-to-use format of the RDT, its deployability in field settings, and its high levels of accuracy lent the test for use in future onchocerciasis elimination efforts (Figure 1).

However, by the time the results of the field studies were published, the study authors had a troubling development to report:

Unfortunately, the Ov-16 card test is no longer available commercially; subsequent to the present study, the manufacturer, AMRAD ICT, went out of business. This is a prime example of the failure of the market to provide critical diagnostic tools for neglected tropical diseases. (Lipner et al., 2006, pp. 220–221)

In 1997, before ICT Diagnostics had even begun work on the onchocerciasis test, the company had been acquired by AMRAD, a research-based pharmaceutical company that had originally been established to help Australian research institutions commercialize their discoveries (Caples & Grace, 2000), for around AU$50 million (Bioworld, 1998), netting ICT’s original investors a substantial profit and giving birth to the short-lived subsidiary AMRAD ICT. In 2000, however, AMRAD got into financial difficulties and was itself bought out by the large US-based diagnostic manufacturer and distributor Binax (Economist, 2000). Against this backdrop, AMRAD ICT was liquidated and the scientific team working on the NTD tests made redundant. It was a devastating blow for Smithyman, who told us:

The last part of this story is that [this] wonderful little company that had almost revolutionized some of these diseases with its rapid tests; it gets shut down, basically just eliminated, and the staff are all made redundant … they paid everyone off and then they had an auction and auctioned off all the equipment and stuff … I remember going. It was a dreadful event for me. It was like going to a funeral.

Following AMRAD’s sale, both the LF and onchocerciasis RDTs developed at ICT Diagnostics came under Binax’s proprietorship. Insofar as the biographies of the LF and onchocerciasis rapid format card tests had closely mirrored one another for several years, their move to Binax marked the point at which their paths sharply diverged.

Binax wasn’t keen on assuming the manufacture of either test, given their perceived lack of profitability and poor alignment with the firm’s existing product portfolio. However, enormous pressure was placed on the firm by the WHO and the newly established Global Programme to Eliminate Lymphatic Filariasis to secure the supply chain of the LF antigen test. In the end, Binax agreed to take on the manufacture of the LF test (commercially available as BinaxNOW Filariasis), which it offered to supply at little more than cost. The deciding factor in agreeing this deal, according to several interviewees, was a guaranteed annual purchase order.^2^

By contrast, a number of factors lined up to work against Binax’s commercialization of the Ov-16 RDT. Although the published scientific evaluations of the test had suggested that it could be used in the elimination of onchocerciasis, no formal goal for eliminating the disease in Africa existed at this time and the proposed use cases were thus entirely hypothetical (Lipner et al., 2006; Weil et al., 2000). Instead, the Onchocerciasis Control Programme was governed by a control goal, which recommended mass drug administration (MDA) of ivermectin medication, supported by two laboratory-based diagnostic approaches: nodule palpation and skin snip microscopy (to look for microfilariae). Both techniques suffered from low sensitivity, but they were still considered to be useful for mapping prevalence in areas of high endemicity in order to determine where MDA should be initiated. Several research laboratories had also developed in-house ELISA tests (a highly sensitive immunological test) for the pathogen, but these had not been validated or standardized, and their accuracy levels were often unclear. Nonetheless, these existing laboratory-based diagnostic approaches were largely deemed adequate for the purposes of disease ‘control’ and there was no audible clamour from the public health community for a new rapid diagnostic test.

The inability to attach the onchocerciasis test to specific global health goals or guidelines meant that neither the WHO nor the Onchocerciasis Control Programme advocated for the Ov-16 RDT’s survival and, in contrast to the LF test, no monetary incentives were being levied to persuade Binax to take the test on. Binax never did go on to commercialize the Ov-16 RDT, and despite Weil and Nutman making several attempts to identify a new manufacturer in the years that followed, the diagnostic test was shelved, effectively becoming the unwanted debris left over from ICT Diagnostics’ multiple acquisitions by larger firms.

This early configurational moment in the history of the Ov-16 RDT reveals the extent to which the perceived commercial value of diagnostic products is dependent on changing social and discursive framings of a disease as a global health priority (Aronowitz, 2008; Leach et al., 2010; Wilkinson, 2017) and the associated construction of diagnostic needs. In this case, the framing of LF as a candidate for elimination assured the test’s manufacturer of the willingness of global health organizations and donors to pay for diagnostic products in the future, even as the continued focus on ‘control’ in the case of onchocerciasis cemented the abandonment of a test that had, from a scientific perspective, performed well in existing validation studies. The stalled career of the ICT Ov-16 test also reveals the extent to which economic valuations of devices tend to increase in salience throughout the product development pipeline, as processes of acquisition and commercial consolidation sideline the original developers of a product along with their more humanitarian aspirations (see also Street, 2023; Rajan, 2003).

## Ov-16 commercialized

In 2010, the African Programme for Onchocerciasis Control (APOC) moved to upgrade onchocerciasis from a ‘control’ to an ‘elimination’ goal, with the disease targeted for elimination—defined as ‘interruption of transmission’—in Africa (Hopkins, 2015; WHO, 2010). As a discursive device, the concept of eradication has substantial conceptual charisma (Packard, 2016; Stepan, 2013). The argument that a disease can be eliminated or eradicated can mobilize support for otherwise neglected causes; it is no accident, therefore, that the emergence of the NTD ‘brand’ (Molyneux, 2012) has coincided with the resurgence of elimination and eradication goals following their brief denigration in the wake of WHO’s failed malaria eradication campaigns in the 1950s (Packard, 2016). For organizations like BMGF, which has thrown its full financial weight behind eradication and elimination agendas (Kelly & Beisel, 2011; Roberts & Enserink, 2007), the setting of bold targets also appealed to a performance-based approach, which values interventions in part for their capacity to generate metrics of success (Adams, 2016). The revitalized perception of elimination as feasible has also been underpinned by renewed technological optimism, and organizations such as the BMGF invest heavily in the development of medical technologies to aid elimination efforts (Packard, 2016; Smith & Taylor, 2013).

Soon after the official reframing of onchocerciasis as a candidate for elimination, Julie Jacobson, then an infectious diseases physician at BMGF, made the case that the Foundation should step in to help onchocerciasis programmes to upgrade their diagnostic toolbox. BMGF considered the current diagnostic landscape, which was limited to clinical diagnosis and procedurally onerous laboratory-based tests, as inadequate to facilitate the more intensive and widespread diagnostic testing that would be needed to meet elimination goals. In part this was because the elimination goal had generated new diagnostic needs. Onchocerciasis control programmes had largely used diagnostic testing to map prevalence of the disease to inform decisions to start MDA in areas where the risk of onchocerciasis was greatest. The elimination goal expanded on this diagnostic scenario to include: mapping areas of low to medium transmission (so called ‘hypo-endemic’ areas); evaluation of the progress toward local elimination of MDA programmes to support decisions to stop MDA; and post-elimination surveillance, to guard against new outbreaks or recrudescence. Within an elimination framework, the purposes of medical testing were extended far beyond its traditional association with clinical management and treatment.

The BMGF first approached US firm Binax to see if it could be persuaded to revive the original AMRAD ICT version of the Ov-16 rapid test. Binax refused. Next, the Foundation contacted Seattle-based non-profit global health organization PATH (formerly known as the Program for Appropriate Technology in Health) to see if it might be interested in developing a grant application to develop another test based on the Ov-16 antigen. Originally established in the mid-1970s as part of the appropriate technology movement, today PATH has become a model for public–private collaboration in the development of global health technologies (Stevenson, 2017) and was for a long time the largest recipient of funding from BMGF, in addition to other donors including bilateral aid agencies (Birn, 2014; Lezaun, 2018).^3^

On the basis of PATH’s non-competitive bid, a grant of US$7,517,087 was awarded by BMGF in November 2010 ‘To make available a rapid, affordable, and field-friendly Ov-16 antigen-based antibody test (“Ov-16 rapid test”) for use in onchocerciasis programs for disease control and eventual elimination in Africa’ (BMGF, 2010). PATH arranged a research and development licence with NIH that allowed Nutman’s group to provide an array of material and technical support to PATH’s team, including a supply of the Ov-16 antigen and standard operating procedures to produce the antigen in-house. Nutman’s lab also identified the secondary antibody to be used in the RDT and tested early prototypes of the antibody test on a selection of contrived and clinical specimens (PATH, 2015a). By this point, NIH’s patent on the Ov-16 protein had lapsed and Binax had no specific IP that would prevent PATH from developing the test. However, Nutman’s group retained valuable expertise and resources, for example clinical specimens on which early prototype performance could be tested, which PATH was keen to benefit from.

Yet while elimination goals helped make the case for the need for novel diagnostic tools in general, they did not in themselves determine the technical characteristics required of such tests. In fact, each elimination use case—whether for mapping of prevalence in endemic areas, evaluating whether to stop MDA, or post-elimination surveillance—had different diagnostic requirements for operability, sensitivity, and specificity. For example, a test to map prevalence as a basis for determining whether MDA should be started would require high levels of sensitivity to ensure that all positive cases were detected since it is the percentage of positive cases that determines whether MDA is appropriate. But when used in a low-prevalence settings, for example as elimination goals are neared, or in post-elimination surveillance, it is important to have high specificity to reduce the chance of a false positive (for complex technical reasons, the proportion of results that are false positives go up as prevalence goes down) (Gass, 2020).

Test design often involves trade-offs and selective optimization. The material constraints of the lateral flow technology utilized by the onchocerciasis RDT, mean that individual tests often need to be optimized for single diagnostic scenarios or ‘use cases’. The upshot is that a single diagnostic test is never going to fulfil all the diagnostic needs for public health interventions identified for a particular disease, or even all the diagnostic needs associated with meeting a single elimination goal. Mapping to support start-MDA decisions, surveillance to support stop-MDA decisions, and post-elimination surveillance all impose different technical requirements, which cannot be met by a single test. As the original developers of the ICT test admitted, for example, optimizing specificity for the test would probably be accompanied by decreased sensitivity (Weil et al., 2000, p. 1798; see also Unnasch et al., 2018, p. i20).

As the project donor, BMGF made the final decision on the use case for which the test should be optimized, and settled on its use to evaluate the success of MDA programmes and to determine that MDA could be stopped in a particular community. The proposed test would use children as a sentinel population to look for active transmission in communities undergoing MDA. Guided by this use case, PATH’s priority became to optimize the RDT for specificity (thus sacrificing sensitivity) so that onchocerciasis programmes wouldn’t encounter false positives that could trigger unnecessary further rounds of MDA. A test calibrated in this way might still show utility in other use cases (for instance, in post-MDA surveillance), but if the test were to be deployed for other purposes its protocol would first need to be carefully revised.

In October 2012, PATH announced its intention to enter into a commercialization agreement with South Korean firm Standard Diagnostics (SD), a subsidiary of Alere, Inc., to manufacture and distribute the Ov-16 rapid test for onchocerciasis (PATH, 2012). SD had already collaborated with the Foundation for Innovative New Diagnostics to commercialize a rapid test for Human African Trypanosomiasis (commercially available as SD BIOLINE HAT) (Taylor & Smith, 2020); SD’s CEO Dr. B. K. Cho described the onchocerciasis collaboration as ‘a valuable extension of our focus to becoming a global leader in the underserved area of neglected tropical diseases’ (PATH, 2012). Tala de los Santos, who was PATH’s Diagnostic Program lead in the years 2012–2019, described to us SD’s strong desire to become a premier diagnostics manufacturer for infectious diseases. From this position, the firm was happy to take on the onchocerciasis rapid test, even though it was unlikely to make them any money (los Santos explained to us that ‘they were hoping to break even to be quite honest’). Even without making profits, the collaboration offered SD two advantages: First, the addition of the Ov-16 test would widen SD’s portfolio—a proven route to building brand equity in the diagnostic business. Second, it provided SD with a strategic partner that was itself well linked to other influential players in global health.

In early 2013, PATH transferred the technology for the onchocerciasis RDT to SD and the latter pursued regulatory approvals and began to prepare for manufacturing. In November 2014, PATH and SD formally launched the SD BIOLINE Onchocerciasis IgG4 rapid test, marketing it as an ‘onchocerciasis surveillance tool’ (PATH, 2015a, 2015b).

The coming into existence of this test can be read as a direct result of the new commercial opportunities afforded the Ov-16 antigen within an elimination framework alongside the growing popularity of donor- and philanthropy-driven product development partnership models. The test’s material characteristics, meanwhile, embodied the efforts by developers and funders to ‘atune’ (Engel, 2020) the technical limitations of a single device to the multiple possible use cases for an Ov-16 test. As we discuss below, this uneven relationship between singular test versus multiple needs proved to be pivotal to the equivocal reception the test received.

## Ov-16 deployed

Almost immediately after its launch, murmurs of discontent with the SD BIOLINE onchocerciasis RDT began to circulate among the global onchocerciasis community. Thomas Unnasch, head of the WHO Collaborating Centre for Onchocerciais Diagnostics, recalled ‘I started hearing stuff right from the very beginning, you know? When they first started sending it out … It was “Well you know, we’re not really sure how well this thing is working”, you’d hear anecdotal stuff.’

Under laboratory conditions, a study had shown the test to have 89.1% sensitivity and 97% specificity (Golden et al., 2013). But testing devices nearly always lose sensitivity in the field and PATH was upfront that ‘additional field validation studies will need to be carried out by multiple partners’ to characterize test performance, to evaluate the feasibility of incorporating the test into existing programmes, and to understand the test’s utility from a programme perspective (Faulx et al., 2014). To assist these validation studies, PATH had developed a standardized ELISA laboratory reference test, against which it could compare the RDT’s performance. Due to a lack of research funding,^4^ rather than run field validation studies itself PATH gave away a first batch of tests to research groups and programmes, funded through an advance market commitment from the Task Force for Global Health (which is itself heavily funded by BMGF, among other public and private donors). Unfortunately, this policy of initial free distribution and of waiting for the evidence base to develop organically proved not only ineffective, but was actively detrimental to perceptions of the test. Implementation partners such as Sightsavers soon began to complain about a drop in the test’s sensitivity in the field.

These concerns were compounded in 2018 when the test was used by the Onchocerciasis Elimination Mapping (OEM) project to assist with the mapping of ivermectin-naïve areas in Ghana and Nigeria (Hamill et al., 2022). While the RDT picked up positive cases in Ghana, alarm bells around its performance started ringing when it picked up almost no positive cases in Nigeria, with the WHO Onchocerciasis Technical Advisory Subgroup noting that, even if there were no cases to be found, ‘given that the specificity of the assay is not 100% we would expect to see some false-positives’ (WHO, 2020a, p. 6).

Data comparing the RDT against a more accurate lab-based ELISA test in two local government areas in Nigeria were presented at the American Society of Tropical Medicine and Hygiene in November 2020 (Igbe et al., 2020). The results showed that the RDT had missed nearly every case picked up by the laboratory reference test—a discrepancy that had serious consequences, potentially making the difference between an area being put forward for treatment with mass drug administration or not.

There is no clear consensus in the onchocerciasis community as to why the SD Bioline test performed poorly in the field (Igbe et al., 2020). One common point made by interviewees was that PATH would have been better to validate and refine the test before it was deployed. However, it was also significant that the laboratory-validated sensitivity of the test was never optimal for the purposes of onchocerciasis mapping, even before it got to the field. The test that BMGF commissioned PATH to develop was optimized for stop-MDA decision making, which required high specificity at the expense of sensitivity. But research groups and onchocerciasis programmes were primarily deploying it for use in mapping to support start-MDA decisions, which favours sensitivity over specificity. The result was that the test was being evaluated by users against a use case for which it had never been designed. The uncertainty that emerged around the public health utility of the test can therefore be viewed as an outcome of the convergence of some degree of technical shortcoming with a moving target around what the test is and is not designed to do.

The ambivalence of the public health community toward the onchocerciasis rapid test shows that ‘success’ in the NTD diagnostics sector does not end with either the development of new tests or their commercialization. While the market launch of the onchocerciasis RDT demonstrated the ability of a partnership model to persuade private industry of the test’s potential commercial value, the value of the device for meeting the needs and goals of disease elimination remained equivocal. This is in part a result of the material affordances of diagnostic technology: Individual devices need to be optimized for specific use case scenarios, but the use cases for which diagnostics are needed are many. When the commercial value of a test is determined by the availability of philanthropic and donor funding rather than the purchasing power of users, and when there is only one diagnostic device in the pipeline, there is a risk that the voices of funders can drown out those of end-users in determining which test gets made.

## Ov-16 unloved

Perhaps surprisingly, its poor performance in the field in Nigeria did not spell the death knell for the SD Bioline RDT. Instead, operational modifications recommended by the WHO Onchocerciasis Technical Advisory Subgroup to change the sample type from whole blood to dry blood spot (DBS) and to move the site of use from the field to the laboratory (WHO, 2020a), helped to improve its operational sensitivity. A new use case consolidated as what Louise Hamill, the technical lead for onchocerciasis at Sightsavers, described to us as an ‘ELISA minimization tool’ for screening samples in the laboratory prior to more accurate ELISA testing, since the RDT is considerably cheaper and easier to perform in a laboratory environment than the ELISA (approx. $US1.25 per sample by RDT versus $US4.70 per sample by ELISA)^5^ (for more costing data, see Hamill et al., 2022). The onchocerciasis community largely saw these modifications as interim, stop-gap measures, while awaiting the development of a new, improved test honed for mapping purposes in the future. The reconfiguration of the RDT as a laboratory-based mapping tool reinvigorated its value for actors involved in the implementation of onchocerciasis elimination programmes and the recommendation to use the RDT as a lab-based tool for mapping purposes, originally an interim measure, has now been normalized and standardized through the WHO’s Onchocerciasis Technical Advisory Subgroup. Nobody we interviewed professed to love the SD Bioline test or extolled its virtues, but with no other rapid tests commercially available to provide valuable data for programmatic decision-making, it was seen as ‘good enough’.

At the same time as public health valuations of the Ov-16 RDT were gradually stabilized around a laboratory-based use case, however, questions began to arise over its commercial viability and longevity. Significant here was the fact that the test had moved, through a series of acquisitions, into the proprietorship of a large, multi-national pharmaceutical company, Abbott. In 2010, another biotech company called Inverness Medical, which later changed its name to Alere Inc., had acquired stakes in SD. In 2017, Alere was itself controversially acquired by pharmaceutical giant Abbott (Balboa, 2017; Nasdaq, 2017). Incidentally, by this point Abbott had also acquired the first iteration of the Ov-16 test, since in 2005 Inverness Medical had also bought Binax, the company that held the rights to the version of the onchocerciasis RDT developed at AMRAD ICT (Tracxn, 2017).

Through these successive mergers and acquisitions, Abbott emerged as market leader in the point-of-care diagnostics market (Nasdaq, 2017). At the same time, and possibly less intentionally, Abbott emerged from the Alere deal with what might be seen as a near monopoly over the manufacture of diagnostic technologies for the NTDs. While SD had intentionally built its brand around a humanitarian niche in global health, Abbott had ended up with proprietorship of the onchocerciasis RDT as a byproduct of its broader acquisitions in the diagnostics arena. By contrast with Binax, Abbott did not shelve the Ov-16 RDT, possibly because a market for the product had already been established among international NGOS and government programmes. However, since Abbott was widely viewed by those in the public health arena as a company with little interest in NTDs, uncertainties remained over Abbott’s long-term commitment to the manufacture of the device.

These uncertainties came to the fore in late spring 2020: As the COVID-19 pandemic grew in severity, rumours began to circulate during the coffee breaks at international conferences and onchocerciasis meetings that Abbott was about to halt the manufacture of the Ov-16 RDT. Some people suspected that Abbott had taken the Ov-16 test off the production line to make way for Covid-19 tests for which there was intense global demand. As one OTS member explained, ‘You didn’t hear it in any of the public meetings or the public sessions at the meetings, it was around the coffee urn: “Have you heard that Abbott’s talking about discontinuing production …?”’ This was of course deeply worrying. While the Ov-16 test was not widely loved, it was recognized as an important component of the slim armoury available with which to fight the disease. The Ov-16 antigen remained the cornerstone of the WHO’s (2016) guidance for stopping MDA and certifying the elimination of onchocerciasis.

As it turned out, manufacture of the test did not halt, and after a short period during which some orders were delayed, which Abbott put down to issues in supply chains caused by COVID-19, regular shipments of the test resumed. Nonetheless, for the organizations that use the test, this brief interlude revealed the continued fragility of a diagnostic infrastructure for onchocerciasis that consists of a single rapid test and a single laboratory ELISA test, and that depends upon a single manufacturer for both.

The source of fragility in the supply of the Ov-16 test matched exactly the source of the test’s equivocal public health value; that is, the narrow use case and, therefore, limited market segment. The test was unloved by users because it only met one out of myriad diagnostic needs. It was unloved by its manufacturers because its limited utility also limited its commercial profitability, especially in the long term and in the face of a changing epidemiological landscape should elimination goals be achieved. Decades of effort, investment, and innovation had resulted in the commercialization of a single rapid test by a single supplier, despite the need for many tests optimized for a diverse range of diagnostic scenarios, and a robust and variegated manufacturing ecosystem for each.

## Conclusion

The Ov-16 RDT is a rare example of the successful commercialization of a diagnostic test for a Neglected Tropical Disease. However the test is seemingly loved neither by the international onchocerciasis community seeking to eliminate the disease, nor by its manufacturer. The test exists in an equivocal state: Its once fluctuating public health purpose has temporarily settled into a narrow use as a laboratory based ‘ELISA minimization’ tool,^6^ which is unlikely to have a major impact on the achievement of elimination goals on its own, while its manufacturers’ views on its economic value and long-term commercial viability remain opaque.

What does this story of an equivocal, unloved test reveal about processes of humanitarian and economic value alignment in global health R&D more widely? First, the story of the onchocerciasis RDT shows that public health valuations of individual medical technologies cannot be taken for granted. Indeed, the focus in much critical global health scholarship on who benefits from the development of global health commodities (e.g., Buse & Harmer, 2004; Graham, 2019; Lazonick & Mazzucato, 2013; Light & Lexchin, 2012; McGoey, 2014; Richter, 2004) and who has access to the resulting products (e.g., Banerjee, 2021; Harman et al., 2021; Sparke & Williams, 2022; Su et al., 2021), can obscure the processes by which particular products come to be deemed public goods at all. Rather than meeting pre-given needs, the onchocerciasis RDT was brought into being well before a public health consensus had emerged around what, precisely, it was for. Elimination targets provided the cornerstone around which such needs could be articulated. However, even once it was agreed that diagnostic tools were necessary to meet elimination goals, the questions of what kind of test, designed for what context of use, remained in flux. And, ultimately, the needs to be met by the commercialized test were themselves ‘innovated’ by programme implementers, who found a new use case for the test in laboratory-based screening. Users’ ongoing work to tinker with the test’s specifications and accuracy through changes in protocol and context of use shows that the design of diagnostic tests and determination of the needs they can meet continues long after products leave the factory floor (Akrich, 1992; Engel, 2020; Hyysalo et al., 2019).

Focusing on global inequities in the distribution of much needed medical products, while important, does not therefore tell the full story of ongoing negotiation over how those needs might be defined. The moral clarity that accompanies the general sense that diagnostics are a global health good becomes much more cloudy once one considers the question of what specific diagnostic tools are for (Kameda et al., 2021, p. 691; Rose, 2013; Sturdy, 2020).

This takes us to a second insight afforded by the biography of the Ov-16 RDT. Why was the test unloved by the onchocerciasis community? The answer to this conundrum lies not in the inadequacy of the test itself, but in the mismatch between the narrow purpose for which the test had been designed—to support stop-MDA decisions—and the broad expectations of how the test could be used—for a wide array of elimination-related use cases—for which its technical qualities had not been optimized.

The continued uncertainty over the specific nature of the Ov-16 RDT’s public health value reveals the fluidity of diagnostic needs in an elimination setting (for example, priorities might change as elimination efforts gather steam and prevalence goes down or up) (Engel, 2020), which poses obvious challenges for commercial viability (Street, 2023), but also, fundamentally, reveals the ‘need’ for multiple different diagnostic tools to serve the multiple purposes of diagnostics in elimination campaigns. In this context, it seems unlikely that a single test product will ever meet the needs of the onchocerciasis community sufficiently to be loved. Our analysis therefore reinforces the finding by other critical scholars of global health, that in practice ‘simple’ technological fixes with narrow applications often fail because of a lack of consideration of the complex social and infrastructural context in which they are embedded (Moran-Thomas, 2015; Packard, 2016). The limited material affordances, and therefore purposes, of individual diagnostics amplify this challenge. However, to understand and address the shortcomings of current technological solutions requires that we go beyond a critique of specific devices to examine the innovation ecosystem within which their development becomes both possible and necessary.

The challenge of meeting multiple and fluctuating diagnostic needs is not unique to the existing global health innovation ecosystem. Nonetheless, the biography of the onchocerciasis RDT suggests that the current innovation ecosystem—which depends on the development of new technologies in small start-ups in the Global North, their acquisition by large pharmaceutical and biotechnology manufacturers, and support for commercialization from public subsidies and philanthropic funding in the form of product development partnerships —is poorly equipped to meet that challenge. The partnerships route to commercialization produces a scattering of isolated diagnostic products, which ultimately end up in the hands of a small number of firms, that often have little interest in the humanitarian or public health value of the tests. Instead, what is needed is an integrated diagnostic infrastructure, consisting of both equipped laboratories and multiple point-of-care tests, capable of meeting diverse and changing programmatic needs.^7^ What, then, might an innovation ecosystem that did account for multiple and fluctuating public health needs look like?

First, if diagnostic needs are multiple and unstable then it is important to ask who gets to define and prioritize innovation for them. A major source of the lack of love for the SD Bioline RDT from within the onchocerciasis community was their lack of involvement in the defining of use cases and original technical specifications for the product, which were determined by donors. A more consensual process for establishing diagnostic needs has been demonstrated more recently in the publication of a set of target product profiles for onchocerciasis diagnostics, produced under the oversight of the WHO Diagnostic Technical Advisory Group (established 2019) (WHO, 2021). The document, which was developed through consultation with implementing partners, Ministry of Health officials and public health researchers, alongside donors and international organizations, is intended as a consensus statement about diagnostic needs for the disease, in addition to providing product developers with a guide as to what kinds of devices are likely to receive public funding for procurement (WHO, 2020b). This document lists five possible use cases for onchocerciasis tests (mapping, monitoring and evaluation, stopping MDA, post-treatment surveillance, and post-elimination surveillance), acknowledging that a single test cannot meet all of these needs—while also establishing agreement among the different actors in the onchocerciasis community around what technical specifications (e.g., sensitivity, specificity, infrastructural, and training requirements) would make a particular diagnostic product good for the two use cases deemed to be the current priority (mapping and stop-MDA decision making). This more inclusive consensus-based approach is a clear improvement on the process by which the priorities for optimization of the SD Bioline RDT were set by BMGF and PATH. The importance of user-led design has also gained increased recognition within the field of biomedical engineering, suggesting that a shift in the norms of global health innovation towards greater democratisation is already in process (Rodriquez et al., 2023).

Second, a focus on the problem of market failure and the need to incentivize private companies in industrialized countries to develop and commercialize diagnostic devices, for example through the writing of target product profiles and establishment of product development partnerships, results in a lack of consideration of the long-term reliable supply of specific products. The process of acquisition and mergers that characterizes the current biotech investment and manufacturing ecosystem makes it more likely than not that diagnostic products will end up in the hands of a small number of pharmaceutical behemoths, where their public health may be of little concern and where they are often relegated to diagnostic debris. Depending on those manufacturers for the long-term production of a test seems naïve, at best.

One suggestion made by interviewees was that we shift to a model of contract manufacturing, where public and philanthropic funds are used to directly procure tests that meet particular product specifications rather than subsidizing companies to make them commercially available. However, this would still depend on a manufacturing ecosystem in which diagnostic tests are made by a small number of manufacturers in industrialized countries far from the point of use. Other models that are gaining in momentum involve efforts to reorient the innovation and manufacturing ecosystem away from centres in the Global North, to invest in biomedical research and development in Africa and systems of decentralized manufacturing and fabrication in the Global South (Fransen et al., 2021; Gomez-Marquez & Hamad-Schifferli, 2019; Juma, 2010). A more equitable approach to global health R&D arguably has the potential to better prioritize the needs of users, ensure more reliable supply chains, and be more flexible to changing diagnostic demands.

Just as the story of the equivocal test might prompt us to rethink how we determine diagnostic needs, so it might prompt us to reimagine the geographies and infrastructures through which new technologies are developed and manufactured. By going beyond a focus on equitable access and distribution on the one hand, and a critique of the technical-fix on the other, to explore what makes technologies ‘good’ for those who make and use them, critical scholars of global health can help prompt global health experts, funders and policy-makers to ask these same questions. The story of the rapid onchocerciasis test shows us that, for global health diagnostics to flourish, rather than becoming diagnostic debris or persisting only as unloved products, requires a fundamental rethink of who does global health innovation and where.

## Notes

1. Since 1987, the global onchocerciasis response has centred on mass treatment of afflicted communities with Mectizan (generic name: ivermectin), a broad-spectrum antibiotic donated by Merck through an early example of public–private partnership, the Mectizan Donation Programme (MDP). Ivermectin is arguably as important in the history of onchocerciasis disease as the Ov-16 protein. Others have told this history of pharmaceutical conjugation (Lezaun, 2018) in more detail (Clemmons, 2008; Collins, 2004; Frost et al., 2002). In this article, we are interested in what the story of the development of an onchocerciasis rapid test can tell us about challenges associated with the development of diagnostic tools for elimination.

2. We were unable to find publicly available documents to corroborate this story.

3. Birn notes that ‘since 1998, Seattle-based PATH (Program for Appropriate Technology in Health), PATH Drug Solutions, and PATH Vaccine Solutions have together received over US$1.6 billion in grants from the BMGF Global Health Program, approximately 15% of global health grants disbursed to date, including close to US$614 million in grants for malaria research; over US$177 million in grants for neglected and infectious diseases; and over US$305 million in grants for enteric diseases and diarrhea’ (Birn, 2014, p. 11).

4. In previous years, the Onchocerciasis Control Programme (1974-2002), had funded numerous operational research projects around promising interventions and tools in collaboration with the WHO’s *Special Programme* for Research and Training in Tropical Diseases. But the budget of the African Programme for Onchocerciasis Control (1995-2015), which had taken over from the Onchocerciasis Control Programme in 2005, did not support an equivalent centralized research programme.

5. These were back-of-the-envelope costings produced by Louise Hamill in 2020, which include shipping (temperature controlled shipping in the case of the ELISA) but not labour costs. New costing data from the OEM pilots have since been published (Hamill et al., 2022).

6. It should be emphasized that this use case is not fixed. PATH have recently published results from their field validation study, suggesting there might yet be a use for the test in non-laboratory settings (Surakat et al., 2023). Since the writing of this article, the Ov16 RDT has continued to be used for some field-based mapping exercises, for example in Tanzania. Although uncertainties over the sensitivity and specificity of the test and issues with reliance on a single supplier were emphasized as shortcomings of the programme (WHO, 2022).

7. Partly in response to the inadequacies of the Ov-16 antibody test, Sightsavers International have made substantial investments in laboratory strengthening in the countries in which they implement onchocerciasis testing.
